# Circular RNA in Pancreatic Cancer: Biogenesis, Mechanism, Function and Clinical Application

**DOI:** 10.7150/ijms.107773

**Published:** 2025-02-28

**Authors:** Hang Chen, Xianxing Wang, Shan Liu, Ziwei Tang, Fuming Xie, Jingyang Yin, Pijiang Sun, Huaizhi Wang

**Affiliations:** 1 Institute of Hepatopancreatobiliary Surgery, Chongqing General Hospital, Chongqing University, Chongqing, 401147, China.; 2 Department of Anesthesiology, Chongqing Seventh People's Hospital, Chongqing University of Technology, Chongqing, 400054, China.; 3 Chongqing Medical University, Chongqing, 400016, China.; Hang Chen and Xianxing Wang contributed equally to this work.

**Keywords:** circRNA, miRNA, pancreatic cancer, biomarker, therapeutic target

## Abstract

Circular RNAs (circRNAs) are a class of novel RNA molecules featured by single-strand covalently closed circular structure, which not only are extensively found in eukaryotes and are highly conserved, but also conduct paramount roles in the occurrence and progression of pancreatic cancer (PC) through diverse mechanisms. As recent studies have demonstrated, circRNAs typically exhibit tissue-specific and cell specific expression patterns, with strong potential as biomarkers for disease diagnosis and prognosis. On the basis of their localization and specific interactions with DNA, RNA, and proteins, circRNAs are considered to possess specific biological functions by acting as microRNA (miRNA) sponges, RNA binding protein (RBP) sponges, transcriptional regulators, molecular scaffolds and translation templates. On that account, further addressing the technical difficulties in the detection and research of circRNAs and filling gaps in their biological knowledge will definitely push ahead this comparatively young research field and bring circRNAs to the forefront of clinical practice. Thus, this review systematically summarizes the biogenesis, function, molecular mechanisms, biomarkers and therapeutic targets of circRNAs in PC.

## 1. Introduction

Pancreatic cancer (PC) is a highly lethal malignancy from digestive system. The 5-year survival rate for PC is only 12%, much lower than for other malignancies 1. In 2024, 66,440 new PC cases and 51,750 deaths are projected to occur in the United States 2. A cross-sectional study reported that PC was predicted to become the second leading cause of cancer mortality in the United States to the year 2040 3. As the detection technology moves ahead speedily, declining trends in various degrees has been observed with regard to the innovation of surgical concepts and techniques, the application of new anti-tumor drugs, and the mortality of most tumors. Nonetheless, the mortality of PC has not been ameliorated and exhibits a slowly upward tendency 2-4. The leading causes behind it may consist in that the non-specific symptoms of PC, the difficulty of early diagnosis, the speedy progression of the disease and the deficiency of treatments 5. For these reasons, it's particularly essential to comprehensively reveal the molecular mechanism of PC occurrence and development, identify specific biomarkers that can be used for early diagnosis and prognosis, and discover new therapeutic targets, which are tremendously crucial to better the diagnosis and prognosis of PC patients.

Circular RNAs (circRNAs) are a class of novel single-stranded RNA molecules characterized by covalent closed rings formed by precursor mRNAs (pre-mRNAs) back-splicing or skipping events of thousands of genes and widely existing in eukaryotes. They are featured by stable structure, high conservation, abundant expression and high endogenous level, and have specific expression patterns in dissimilar tissues at various developmental stages 6. In 1976, Sanger *et al.* discovered the first circRNA molecule in viroid through electron microscopy 7. As already indicated by early studies, only a few circRNAs (such as circSRY, circDCC, and circEST1) have biological functions, while most circRNAs are merely "by-products" or "garbage" produced by abnormal splicing 8. In recent years, as high-throughput RNA sequencing, circRNA-specific microarrays and bioinformatics advance speedily, a multitude of circRNAs have been discovered and identified. Aside from that, their cell/tissue and time-specific expression patterns and functions have been revealed in an all-round manner 9. On the basis of genomic origin and biogenetic pattern, circRNAs are currently categorized into circular intron RNAs (ciRNAs), exon-intron circRNAs (EIcircRNAs), exon circRNAs (EcircRNAs) and tRNA intronic circular RNAs (tricRNAs) 10. As suggested by multiplying evidence, circRNAs conduct a key regulatory role in tissue development, aging and disease occurrence (such as tumors, neurological diseases, diabetes, cardiovascular diseases and chronic inflammatory diseases) 11. In particular, the role of circRNAs in cancer occurrence and progression has captured massive attention. More importantly, they possess the potential to serve as cancer biomarkers and new therapeutic targets 12-15. As already illustrated by further studies, circRNAs conduct paramount roles in cancer initiation and development through multiple molecular mechanisms, such as regulating gene transcription 9, acting as miRNA sponges 16-18, interacting with RNA binding proteins (RBPs) 19, 20, and translating into proteins/peptides 21. Consequently, further understanding of the biogenesis, functions and mechanisms of circRNAs in PC may provide brand new references and ideas with respect to the exploration of potential molecular diagnostic markers and therapeutic targets in PC.

## 2. Biogenesis

**Figure 1:**
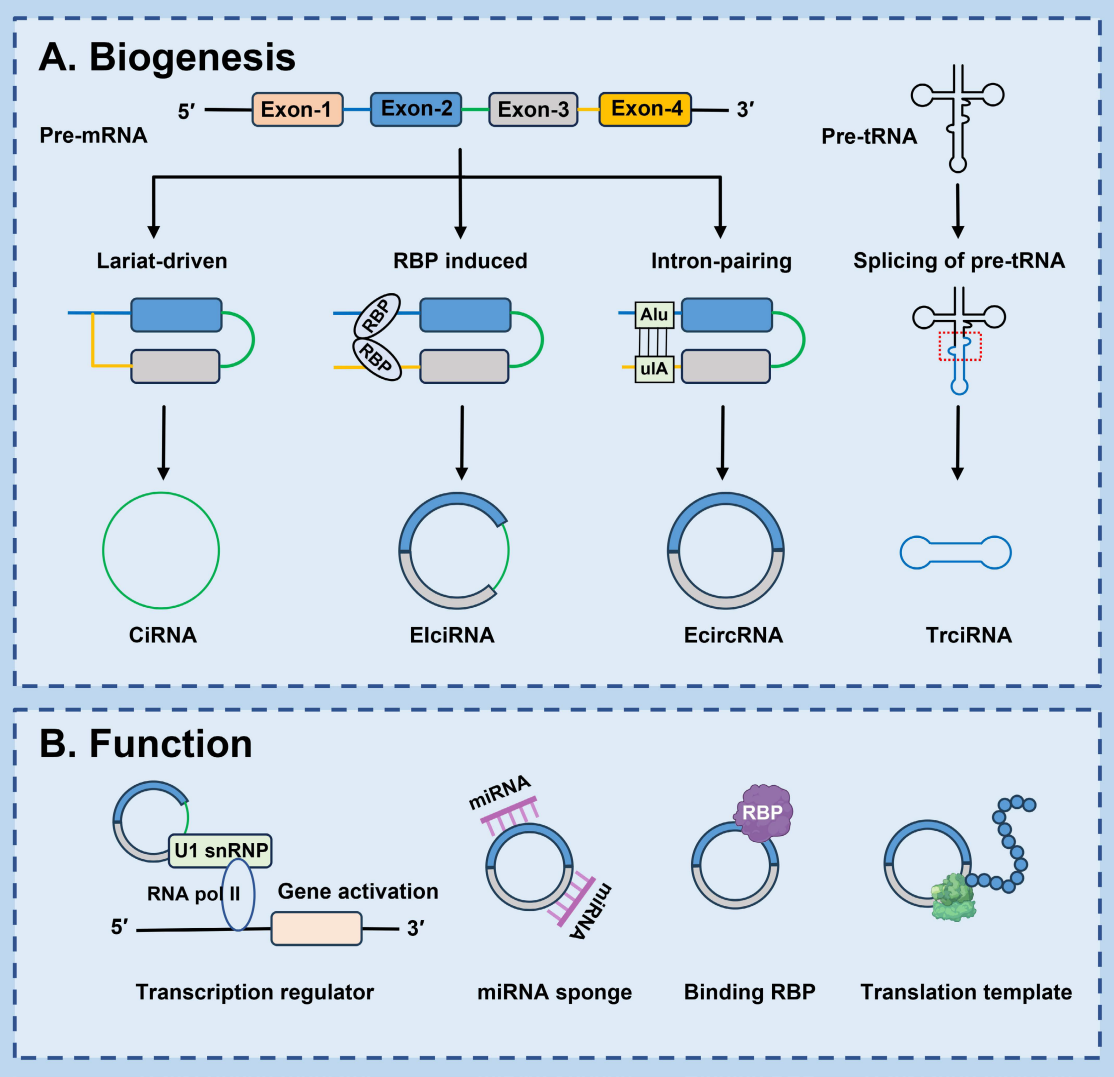
** Biosynthesis and functions of circRNAs. (A) Lariat-driven circularization:** Pre-mRNA can produce a lariat intermediate by exon-skipping. The intron sequences in the lariat intermediate are retained, and then form ciRNA; **RBP induced circularization:** RBPs specifically bind to specific sites in the flanking intron sequence of pre-mRNA to form a bridge, thereby increasing the formation of EIciRNA; **Intron-pairing-driven circularization:** The upstream introns are base-paired with the downstream introns, forming EcircRNA; **Splicing of pre-tRNA-driven circularization:** Pre-tRNA is cleaved into half of the exon and intron part. TricRNA is produced by joining the termini of the introns. **(B) Transcription regulator:** CircRNAs can interact with U1 small nuclear ribonucleoproteins (U1 snRNPs) or RNA polymerase II (Pol II) in the promoter region of targeted genes, and enhance the transcription and splicing of genes; **microRNA (miRNA) sponge:** CircRNAs with complementary binding sites can pairing with miRNAs response elements of mRNAs and sequester miRNAs away from target mRNAs, thereby protecting target mRNAs from miRNA-dependent degradation; **Binding RBP:** CircRNAs may act as sponge, protein scaffold, or decoy for proteins directly or indirectly regulate their locations and functions; **Translation template:** CircRNAs may be translated into unique peptide by cap-independent translation initiation mechanisms under specifical conditions.

The biogenesis of circRNAs apparently differs from the production mechanism of mRNA. Back-splicing of pre-mRNA is the predominant process for circRNA generation. A splice donor that is downstream of the 5' splice site is joined to a splice acceptor that is upstream of the 3' splice site, producing a circular format with a 3'-5' phosphodiester bond at the back-splicing junction site 22. Currently, the common models of circRNA circularization include: lariat-driven circularization, RBP induced circularization, intron-pairing-driven circularization and splicing of pre-tRNA-driven circularization 10 (Fig. 1).

### 2.1 Lariat-driven circularization

Pre-mRNAs are partially folded as a result of exon hopping during transcription. The upstream 3' splicing receptor is covalently linked to the downstream 5' splicing donor to form a lariat intermediate containing exons or introns. Subsequently, the lariat intermediate undergoes reverse splicing, excision or retention of the sequence, and eventually forms a covalently closed ciRNAs 23. In addition, ciRNA formation depends on conserved elements which are seven nucleotides rich in GU near the 5' splicing site and 11 nucleotides rich in C near the 3' branching point. In the process of reverse splicing, these two elements combine to form a lariat structure, and then the exons in the structure are removed by splicing. These two elements protect ciRNA from intron debranching and degradation, ultimately forming a stable ciRNA 24, 25.

### 2.2 Intron pairing-driven circularization

In this process, introns on either side of exons are essential elements. The upstream introns and the downstream introns contain reverse complementary sequences (such as Alu repeats and other nonrepetitive elements), after base pairing occurs, the downstream 5-donor and upstream 3-receptor splicing sites are close to each other, ultimately speeding up ecircRNAs formation 26, 27. Some bioinformatic analyses and experimental studies have also confirmed that reverse complementary Alu repeats in flanking introns are associated with circRNA biogenesis 28. Li *et al.* demonstrated that the sequences of flanking introns of cGGNBP2 (hsa_circ_0003930) was highly reversed complementary and necessary for the circRNA biogenesis 29. What's more, circHIPK3 expression is promoted by Alu elements 30.

### 2.3 RBP-driven circularization

CircRNA biogenesis is regulated by a variety of proteins, such as RBPs, enzymes, and transcription factors 31-34. RBPs bind specifically to specific sites in the flanking intron sequence of pre-mRNA to form a bridge, making the splicing sites closer between the receptor and donor. In the process of back splicing, some intron sequences are not removed but remain in the circRNA, known as EIcircRNAs 35. For instance, mannan binding lectin (MBL) heightens circMBL generation by recognizing and binding highly conserved MBL-binding elements located in the flanking introns of its own pre-mRNA 36. Fused in sarcoma binds to RHOBTB3 pre-mRNA and then accelerates the back-splicing process of circRHOBTB3 production 32. Moreover, quaking, acting as an alternative splicing factor 37, induces exon cyclization by binding with the recognition element in the intron and forming a dimer, and subsequently gives rise to the generation of circRNA, which also facilitates the reverse splicing efficiency in the epithelial-mesenchymal transition (EMT) process 38. Apart from that, adenosine deaminase 1, as an RNA editing enzyme, negatively regulates the production of circRNA by lessening flanking introns and reverse folded RNA pairing structures 33. Likewise, the RNA/DNA helicase DExH-Box helicase 9 lessens circRNA formation by down-regulating intron pairing 39-41.

### 2.4 Splicing of pre-tRNA-driven circularization

Additionally, a subset of circRNAs is derived from tRNAs through the splicing of pre-tRNAs 42. In the process of tRNA maturation, the tRNA splicing endonuclease complex cleaves pre-tRNAs, and an enzyme known as RNA terminal phosphorylase B (RtcB) ligates the exon fragments and introns to generate a tricRNA 43, 44.

To put it simple, the biogenesis of circRNAs and the molecular mechanism that remain inconclusive with regard to how they regulate circularization. It is noteworthy that the origin of circRNA is closely related to parental genes. Currently, the genetic origin of circRNA can be queried through a series of databases, which will drive us to gain in-depth insight into the circularization process of circRNAs (Table 1).

## 3. Mechanisms of circRNAs in PC

### 3.1 MiRNA sponges

As ubiquitous and conserved small non-coding RNAs with lengths of 19-25 nucleotides, miRNAs prevent translation or facilitate degradation by binding to specific miRNA responsive element of target mRNA. In such case, it thereby exhibits a wide range of biological functions 45. On the basis of the available evidence, the researchers define this class of circRNA with miRNA-bound miRNA response element (MRE) as competitive endogenous RNA (ceRNA). By acting as miRNA molecular sponges, these circRNAs bind to corresponding miRNAs and inhibit their functions, and regulate the translation or degradation of target molecules, thus exerting tumor inhibition or carcinogenic effects 46, 47. As Multiple studies have confirmed that circRNAs can act as miRNA sponges in PC (Supplementary Table 1). For instance, circ001859, circSEC24A, circMBOAT2, circRNF13 and circ000684 affect malignant phenotypes of pancreatic ductal adenocarcinoma (PDAC) cells via sponging dissimilar miRNAs 80, 83, 117, 120, 123. Likewise, circATG7 and circRHOBTB3 sponges miR-766-5p and miR-600 to regulate the autophagy response of PDAC cells, respectively 19, 32. Apart from that, hsa_circRNA_001587 and circ_0000284 regulate angiogenesis of PC cells via binding to miR-223 and miR-1179, respectively 124, 125. As illustrated by other studies, circ_0058058 acted as a molecular sponge of miR-557 to mediate PC immunosuppressive microenvironments 133. A myriad of evidence confirmed that circRNAs can function as miRNA sponges. Nonetheless, not all circRNAs possess this capability, which is owing to the fundamental fact that some specific circRNAs have little or even no MRE.

### 3.2 Binding RBPs

RBP is a group of proteins with RNA recognition and binding ability, which conducts an indispensable role in biological processes by binding RNA 135. As already revealed by recent studies, circRNAs may bind to RBPs to regulate the expressions of core genes in the process of PC (Supplementary Table 1). Meng *et al.* suggested that circSTX6 interacted with cullin-2 (CUL2) to participate in the ubiquitin-dependent degradation of hypoxia-inducible factor 1-alpha (HIF1A) and subsequently accelerating PDAC progression 79. Another study demonstrated that circRTN4 interacted with EMT-driver RAB11FIP1 and blocked its ubiquitination site to lower its ubiquitination in PDAC 78. Furthermore, circPTPN22 attenuates signal transducer and activator of transcription 3 (STAT3)/sirtuin 1 (SIRT1) interaction by binding to STAT3, ultimately facilitating STAT3 acetylation during PC immune resistance 128. Other investigations have revealed that circMYO1C targets the m6A site of programmed death-ligand 1 (PD-L1) mRNA to reinforce its stability through cooperating with insulin-like growth factor 2 mRNA binding protein 2 (IGF2BP2), thereby pushing ahead PDAC immune escape 130. As mentioned above, circRNAs can bind to RBPs to facilitate or inhibit their functions. Nevertheless, rare further studies have been found to probe deep into the interactions between circRNAs and RBPs in PC. For this reason, RBP immunoprecipitation combined with circRNA sequencing can be adopted to discover circRNAs that can interact with RBPs.

### 3.3 Protein scaffolds

As displayed by new evidence, circRNAs can also serve as protein scaffolds, interacting with proteins to regulate protein structures or functions, thereby participating in pathological and physiological processes (Supplementary Table 1). One study illustrated that circEIF3I was a molecular scaffold that interacted with small mother against decapentaplegic family member 3 and adaptor-related protein complex 2 subunit alpha 1 to form a ternary complex, which immensely facilitated growth and metastasis of PDAC 20. Furthermore, circATG7 act as a scaffold to increase the interaction between the human antigen R protein and ATG7 mRNA as well as reinforce ATG mRNA stability in PC cells 19. To go it further, other research has demonstrated that circPTPN22 regulates PC immune microenvironment by facilitating STAT3 acetylation via inhibiting STAT3/SIRT1 interaction 128. More importantly, circRNAs can also conduct biological functions as the molecular scaffold while functioning as sponges for miRNAs. CircFOXK2 facilitates growth and metastasis of PDAC by complexing with Y-box-binding protein 1 (YBX1) and heterogeneous nuclear ribonucleoprotein K and sponging miR-942 52. Likewise, exosomal circPDK1 sponges miR-628-3p to activate the bromodomain plant homeodomain finger transcription factor (BPTF)/c-myc axis and serves as a scaffold to strengthen the interaction between ubiquitin-conjugating enzyme E2 O and bridging integrator 1 (BIN1), which gives rise to PC glycolysis 111. Altogether, all of these significant findings evidently reveal that circRNAs can act as protein scaffolds to affect the expression or function of certain proteins, which expands our understanding of circRNAs. But it remains essential to look into the specific mechanisms by further exploration.

### 3.4 Regulators of gene splicing and transcription

In general, the majority of EIciRNAs and ciRNAs are located in the nucleus, thus possessing the ability to exert regulatory effects at the transcriptional or post-transcriptional level 136. Associated research has illustrated that circ-EIF3J and circ-PAIP2, identified as EIcircRNA, can combine with snRNP to form EIciRNA-U1 snRNP compounds, which further interact with Pol II to facilitate the transcription of EIciRNA parent genes 137. Additionally, the specific interaction between ci-ANKRD52 and RNA Pol II complex facilitates ankyrin repeat domain 52 transcription 138. It is particularly noteworthy that alternative splicing is extensively correlated with a variety of biological processes. Furthermore, circRNAs also participate in the regulation of gene expression by affecting pre-mRNA alternative splicing. For instance, circMBL competes with the classical splicing of MBL pre-mRNA, thereby weakening the formation of linear RNA 36. Furthermore, hsa_circ_0007919 recruits forkhead box protein A1 and ten eleven translocation 1 to lessen the methylation of the ligase I promoter and heighten its transcription, which tremendously facilitate the repair of base excision, mismatch and nucleotide excision 102. Likewise, Kong *et al.* uncovered that circ-CDYL cooperated with a novel transcriptional (co-) factor eukaryotic elongation factor 1alpha-2 to initiate collagen, type XIV, alpha1 transcription 139. Another study investigating breast cancer has proved that antisense circular RNA circSCRIB hindered the splicing of scribble (SCRIB) pre-mRNA, and interacted with SCRIB mRNA sequence, which resulted in the inhibition of SCRIB translation 140. As jointly revealed by these significant findings, circRNAs affect gene expression at the transcriptional or post-transcriptional level. Nevertheless, the current research on the transcriptional regulation and splicing of circRNA in PC is relatively lagging behind that in other tumors, which apparently necessitates more comprehensive exploration and more conspicuous reinforcement.

### 3.5 Protein/peptide templates

Early studies have illustrated that circRNAs, as non-coding RNAs, cannot be translated through a cap-dependent mechanism owing to the deficiency of 5' end and 3' Poly A tail 141. Nonetheless, as the study deepens its depth and width, some circRNAs with initial codon sites, ORF and IRES elements are translated in a cap-independent manner in some cases, producing proteins or peptides with specific functions 142-144. For instance, circSEMA4B which contains AUG, ORF and IRES encodes a novel protein SEMA4B-211aa. SEMA4B-211aa inhibits the production of phosphatidylinositol 3,4,5-trisphosphate by binding to p85, thus inhibiting the phosphorylation of protein kinase B (AKT) (Thr308), and ultimately inhibiting the progression of breast cancer 145. As clearly proved by another study with regard to colorectal cancer, a spanning junction ORF with the potential to encode a 121 amino acid protein and an IRES at 207-292 nt are contained in the sequence of circINSIG1. circINSIG1 encods a 121 amino acid protein circINSIG1-121 to accelerate cholesterol metabolism in cancer 146. Apart from IRES and ORF responsible for circRNA translation, the m6A modification can also initiate or reinforce circRNA translation 147. The m6A reader YT521-B homology domain 2 drives the hsa_circ_0082002 encoding the protein MET404 in glioblastoma 148. Nonetheless, no reports have been released to investigate circRNA encoding protein in PC. It is also noteworthy that circRNA translation is inadequate. Worse still, controversial debates are still heating with respect to how its derived proteins or peptides affect PC development. As a consequence, more information is essential to elucidate the mechanisms of circRNA translation and deepen our understanding of the circRNA molecular mechanisms and human proteins.

## 4. Functions of circRNAs in PC

### 4.1 Proliferation and tumorigenesis

**Figure 2:**
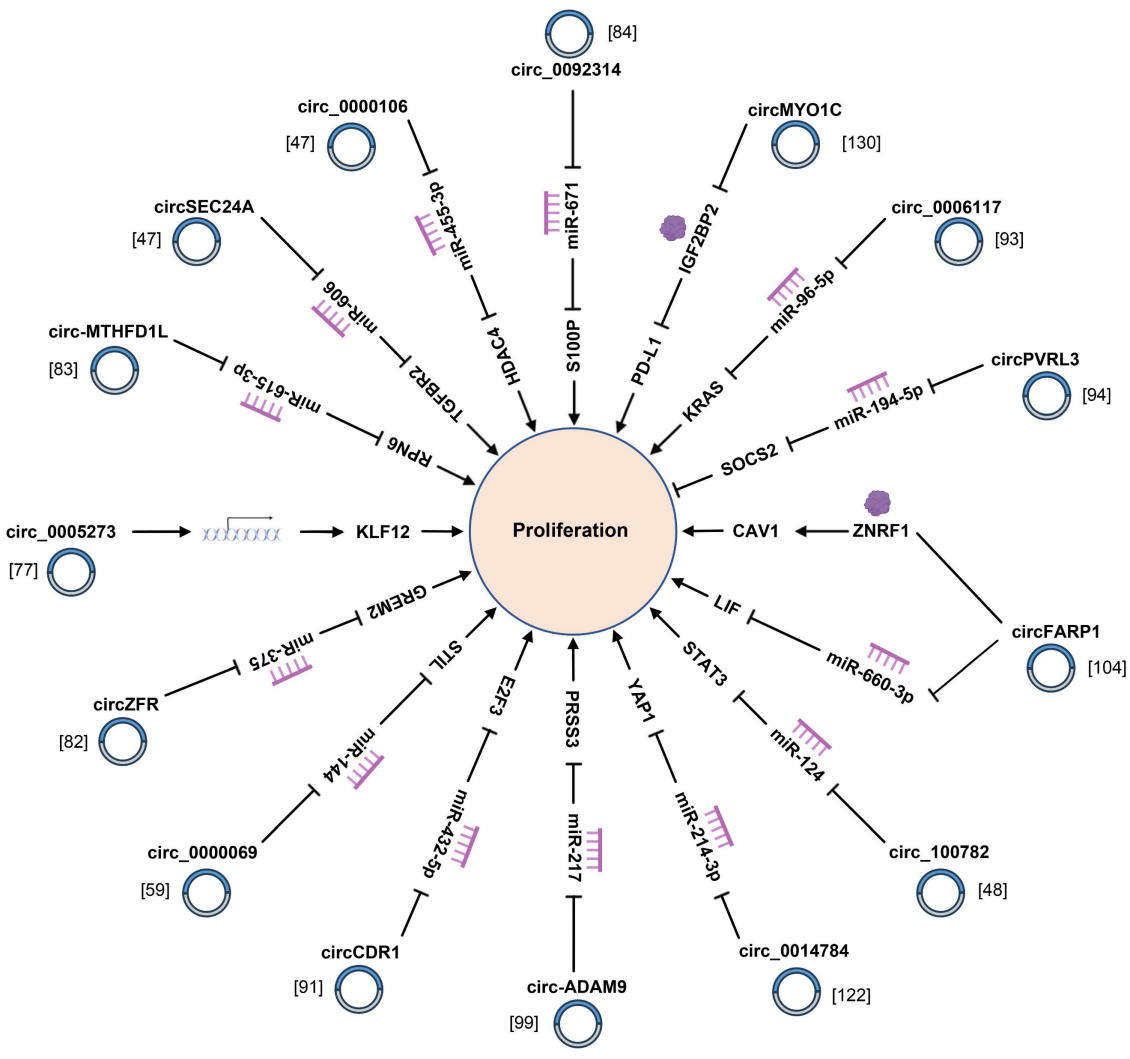
CircRNAs affect the proliferation of pancreatic cancer cells via regulating miRNA-mRNA axis.

**Figure 3:**
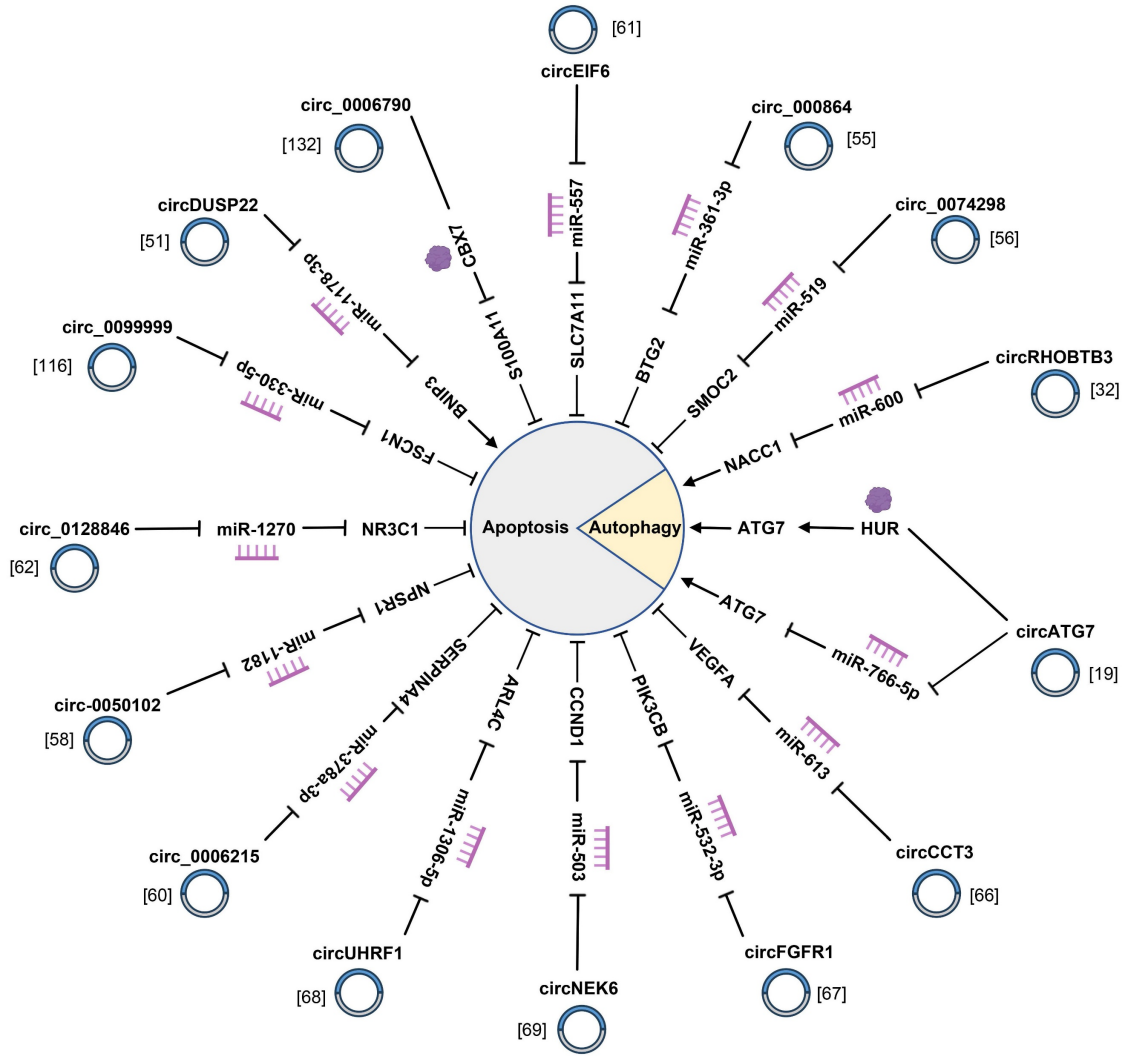
CircRNAs are involved in the apoptosis and autophagy of pancreatic cancer cells by interacting with miRNAs and RNA-binding proteins.

Normally speaking, cancer cells are featured by continuous proliferation and avoidance of apoptosis 149. These key cellular processes are regulated by multiple factors that have not yet been sufficiently elucidated. As already demonstrated by relevant studies, multiple circRNAs with dysregulated expression in PC tissues and cells are tightly correlated with proliferation and apoptosis of PC cells (Fig. 2, 3) (Supplementary Table 1). For instance, circ-MBOAT2 regulates tumor growth and apoptosis via miR-433-3p/ glutamic-oxaloacetic transaminase 1 signaling axis in PC 117. Li *et al.* reported on exosomal circ_0030167 derived from bone marrow mesenchymal stem cells (BM-MSCs) implicated in proliferation, and stemness of PC cells via the miR-338-5p/wingless-type MMTV integration site family (WNT) inhibitory factor-1/Wnt8/β-catenin axis 119. Moreover, circSFMBT1 affects proliferation and apoptosis of PC cells by augmenting the mRNA level of p21-regulated kinase 1 54. As revealed by a recent study, hsa_circ_0006790 is involved in PC cells proliferation and apoptosis by cooperating with chromobox protein homologue 7 132. Furthermore, circRNA_102049 interacts with miR-455-3p to accelerate CD80 transcription, thereby inhibiting PC proliferation and attenuating apoptosis 134. hsa_circ_0000069 facilitates the proliferation and cell cycle progression of PC cells by suppressing the expression of the parent gene stem cell leukemia/T-cell acute lymphoblastic leukemia 1 interrupting locus for hsa_circ_0000069 59. To further clarify the indispensable role of circRNAs in proliferation, apoptosis, autophagy and tumorigenesis of PC cells is advantageous for deepening the understanding on the pathogenesis of PC.

### 4.2 Metastasis

**Figure 4:**
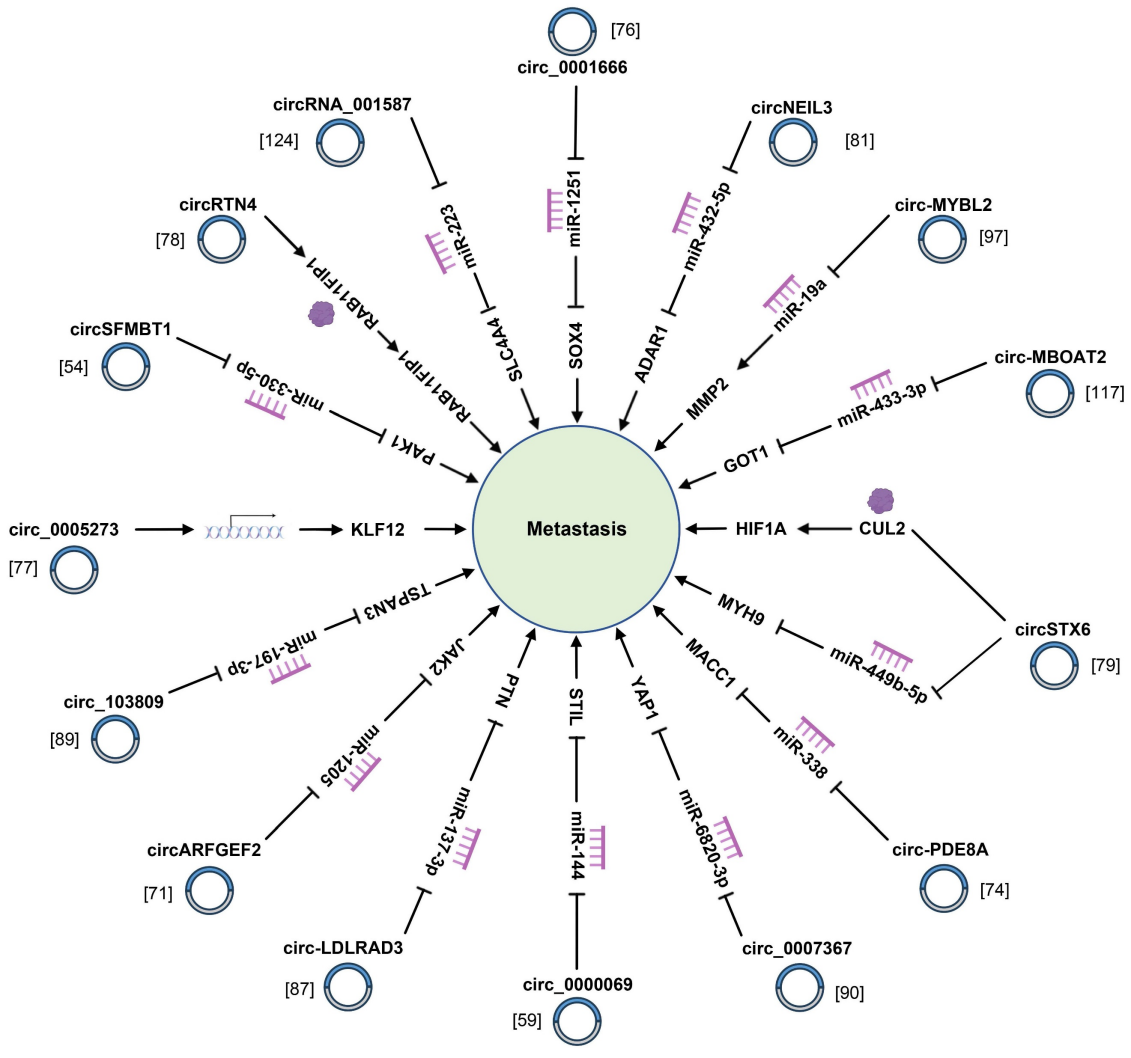
CircRNAs modulate the metastasis of pancreatic cancer cells through sponging miRNAs and binding to related proteins.

Aside from the infinite growth of tumor cells, metastasis is another pivotal feature of tumor cells. As revealed by recent studies, some circRNA have close correlations with tumor metastasis (Fig. 4) (Supplementary Table 1). For instance, circ_0000284 is remarkably upregulated in PC tissues and cells, and facilitates PC cell proliferation, migration, and invasion while regulating apoptosis 125. Besides, hsa_circRNA_001859 acts as a molecular sponge of miR-21-5p and regulates the expression of solute carrier family 38 member A2 to regulate tumor EMT of PC 80. Additionally, circ-STK39 regulates translocation-associated membrane protein 2-mediated proliferation and the EMT of PC by sponging miR-140-3p 85. Chen *et al.* identified that circSEC24A noticeably governed the proliferative, migration and invasive capacity of PC cells through accelerating the expression of TGF-beta receptor 2 (TGFBR2) 83. Apart from ordinary circRNAs, exosomal circRNAs also conduct a pivotal role in the process of PC cells. For instance, exosomal circPDK1 strikingly facilitates PC cells migration, proliferation, and glycolysis by modulating the miR-628-3p/BPTF axis and degrading BIN1 111. Another study suggested that exosomal circ_0030167 inhibited the invasion, migration, proliferation and stemness of PC cells by inactivating the Wnt/β-catenin signal pathway 119. A great many clinical practices have confirmed that tumor metastasis greatly affects the diagnosis and treatment of PC, so more studies are needed to understand the role of circRNAs in PC metastasis.

### 4.3 Chemotherapy resistance

**Figure 5:**
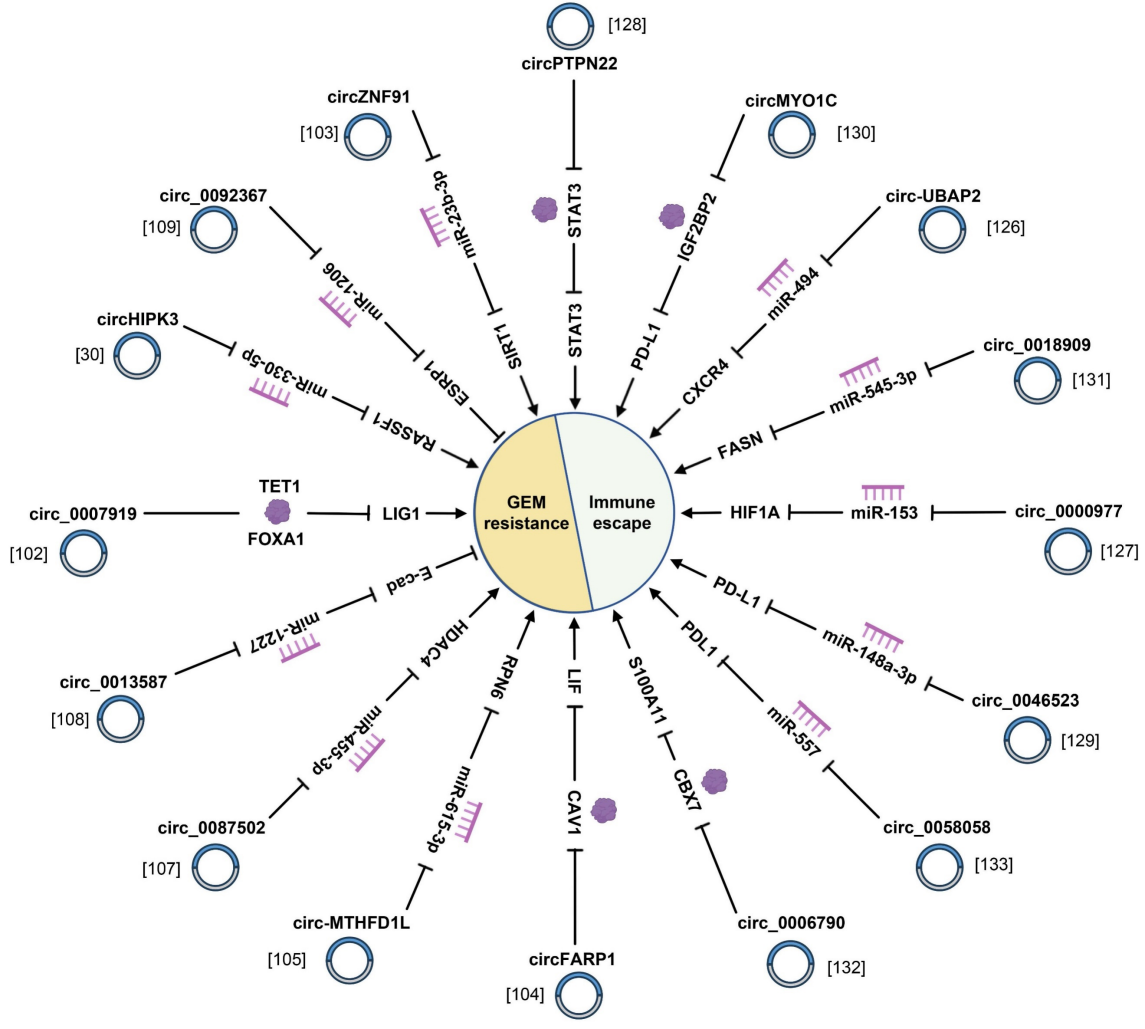
CircRNAs play crucial roles in GEM resistance and immune escape of pancreatic cancer.

Chemotherapy is one of the predominant treatments for PC. Nevertheless, the emergence of chemotherapy resistance and multi-drug resistance seriously affects the effectiveness of PC treatment and is one of the crucial factors bringing about undesirable prognosis of PC patients 150. In recent years, as the study probes deeper into circRNAs, a new perspective has been provided for people to understand the mechanism of drug resistance in PC (Fig. 5) (Supplementary Table 1). For instance, circ_0087502 gives rise to PC cell proliferation, migration, and gemcitabine (GEM) resistance by sponging miR-1179 and facilitating TGFBR2 expression 107. Moreover, cancer-associated fibroblast (CAF)-specific circFARP1 inhibits caveolin-1 degradation and enhances leukaemia inhibitory factor (LIF) secretion, which ultimately triggers GEM chemoresistance in PDAC 104. As suggested in the studies conducted by Chen *et al.*, circMTHFD1L, as an endogenous miR-615-3p sponge, upregulated the expression of ribophorin VI, which not only accelerated the repair of DNA damage, but also noticeably weakened the sensitivity of PC to GEM 105. Another study suggested that circHIPK3 accelerated GEM resistance in PC cells by targeting Ras-association domain family 1 via miR-330-5p 30. The above circRNAs conduct a catalytic role in GEM resistance, while some circRNAs induce PC cells to be sensitive to GEM. hsa_circ_0007401 and circ_0092367 augment the chemosensitivity of GEM in PC 101, 109. What's critical to mention is that circRNAs not only regulate GEM resistance of PC cells, but also affect therapeutic efficacy of the small-molecule epidermal growth factor receptor tyrosine kinase inhibitor against PC 108. On the whole, these studies have persuasively illustrated that circRNAs conduct irreplaceable roles in modulating chemosensitivity in PC. Hence, delving deeply into the potential roles of circRNAs in the mechanism underlying drug resistance in PC is advantageous for the discovery of novel molecular targets and the enhancement of chemotherapy efficacy, which is of paramount value for the prognosis of patients with PC.

### 4.4 Immune escape

Tumor-related immunity can exert profound influence in tumor progression and treatment. As reported by recent studies, circRNAs are prevalently involved in tumor immune escape (Fig. 5) (Supplementary Table 1). For instance, hsa_circ_0006790 is highly expressed in PC and is involved in immune escape 132. Furthermore, circMYO1C mediated by m6A methyltransferase methyltransferase like 3 targets PD-L1 mRNA stability by cooperating with IGF2BP2, thereby accelerating PDAC immune escape 130. Another study suggested that circPTPN22 could trigger the formation of PC immune microenvironment by facilitating STAT3 acetylation via attenuating STAT3/SIRT1 interaction 128. Moreover, circ_0058058 exhibits elevated expression in PC tissues and works as a molecular sponge of miR-557 to upregulate PD-L1, thereby resulting in PC progression and immune escape 133. Aside from cancer cells, other cells can also exert certain influence in the formation of tumor immune microenvironment, such as CAFs, macrophages, and T cells. Fu *et al.* reported that hsa_circ_0046523 was beneficial for an immunosuppressive tumor microenvironment by propelling the apoptosis and exhaustion of CD8+ T cells, inhibiting CD8+ T cell function, lowering the secretion of immunosuppressive cytokines interleukin-10 and TGF-β, and lessening the secretion of immune effector cytokines interferon-gamma and IL-2 among among peripheral blood mononuclear cells 129. Additionally, circCUL2 is specifically expressed in CAFs and induces the inflammatory CAF (iCAF) phenotype, subsequently iCAFs facilitates PDAC progression through IL-6 secretion 100. Other than that, a study on circ_0018909 illustrated that circ_0018909 induced polarization of M0 macrophages to M2 macrophages to regulate the development of PC 131. With the belief that studies about circRNAs play a pivotal role in PC-related immunity, and more far-reaching research on the mechanism of circRNAs in immune regulation is advantageous to explore a new breakthrough in the treatment of PC.

### 4.5 Glycolysis

**Figure 6:**
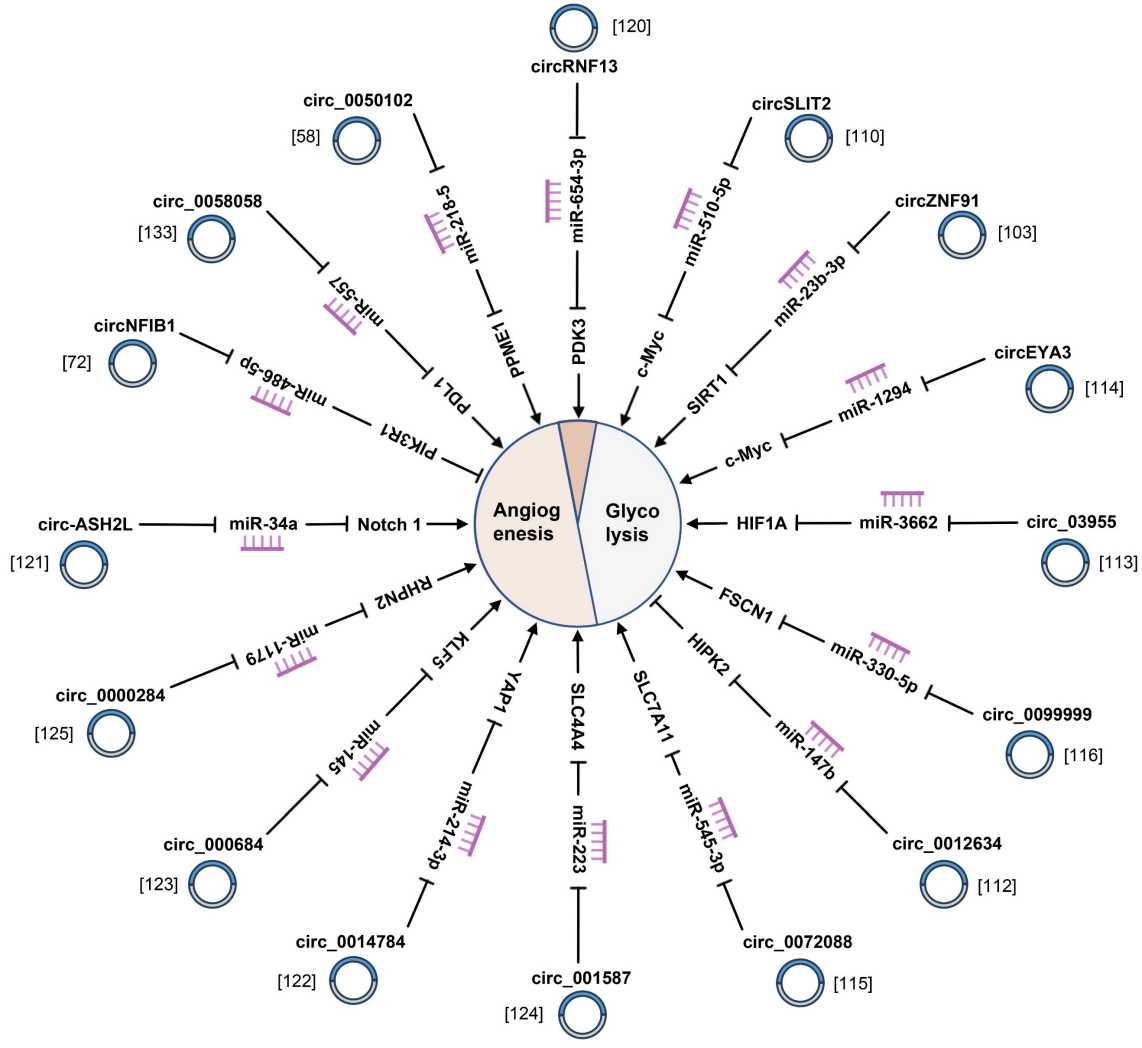
CircRNAs are responsible for angiogenesis and glycolysis of pancreatic cancer.

Most solid tumors are typically characterized by the hypoxic microenvironment which can be attributed to the speedy proliferation of tumor cells, heteromorphic tumor structure, and aberrant structure and function of tumor vessels. During this process, cancer cells obtain energy through glycolysis under hypoxia 151. CircRNAs are associated with hypoxia (Fig. 6) (Supplementary Table 1). Zhao *et al.* reported that circRNF13 induced by hypoxia was upregulated in PC tissues and accelerated tumor glycolysis in PC 120. Moreover, circ_0072088 facilitates cell extracellular acidification rate, lactate production, glucose uptake, and ATP generation, but suppressed oxygen consumption rate in PDAC cells 115. Furthermore, circ_03955 regulates Warburg effect of PC by the promotion of HIF-1ɑ 113. Simultaneously, circSLIT2 can propel the aerobic glycolysis of PDAC via targeting miR-510-5p/c-Myc/ lactate dehydrogenase A axis 110. These significant findings reveal that to look into how circRNAs affect anaerobic and aerobic glycolysis is likely to provide a novel sight for PC treatment.

### 4.6 Angiogenesis

Angiogenesis, the formation of new blood vessels from pre-existing ones, is an essential process for growth, development and disorders. Angiogenesis is bound up with tumors, which can be certified by the extensive application of antiangiogenic drug bevacizumab in anti-cancer 152. Nevertheless, no antiangiogenic drug has been developed to treat PC. As evidently demonstrated by numerous studies, circRNAs can impose remarkable effects on angiogenesis of PC cells (Fig. 6) (Supplementary Table 1). What should be pointed out is that hsa_circRNA_001587 inhibits angiogenesis in PC by impairing miR-223-mediated solute carrier family 4 member 4 inhibition 124. Furthermore, circ_0000284 facilitates PC angiogenesis resting with the regulation of miR-1179/rhophilin 2 125. Meanwhile, hsa_circ_0050102 expedites angiogenesis of PC through growing phosphatase methylesterase 1 abundance 58. Thus, we provide an all-round overview of recent insights into circRNAs in blood vessel development, which will present brand new opportunities and enlightening references for the design of vascular-directed therapies in PC.

### 4.7 Other

PC has the marked desmoplastic reaction. As the dominant cell type within the tumor stroma, CAFs critically contribute to biological behaviors in this highly fibrotic solid malignancy 153. Recently, Rong *et al.* reported that circBIRC6, a CAF-derived extracellular vesicles-packaged circRNA, induced oxaliplatin resistance by mediating SUMOylation of X-ray repair cross-complementing protein 4 154. Moreover, Chen team elucidated two CAF-derived circRNAs. circCUL2 was significantly correlated with the poor survival of PDAC patients. circCUL2 induced normal fibroblasts to transformed into iCAF phenotype, and then secreted IL-6 to activate STAT3 signaling pathway in cancer cells, ultimately inducing PDAC progression 100. Furthermore, a CAF-specific circFARP1 is positively correlated with poor survival in advanced PDAC patients. circFARP1 interacts with caveolin 1 and miR-660-3p, enhancing LIF secretion to facilitate GEM resistance and tumor cell stemness 104. Significantly, cancer stem cells are important cells that play a fundamental role in cancer 155. The roles of PC-associated circRNAs in cancer stem cells has also received attention. circRREB1 is significantly upregulated in PDAC and promotes WNT7B transcription by directly interacting with YBX1 and facilitating its nuclear translocation, consequently activating the Wnt/β-catenin signaling pathway to maintain PDAC stemness 118. At present, there is still a lack of research on circRNA about CAF and stem cells in PC. To further clarify the biological functions and molecular mechanisms of circRNAs in CAF differentiation and cancer stem cells will help promote the progress of circRNA research.

## 5. Clinical application of circRNAs in PC

### 5.1 Diagnostic and prognostic markers

Early detection, diagnosis and treatment are of vital importance in improving the prognosis of cancer patients. Nevertheless, inappropriate prognostic evaluation will seriously affect the adjustment of treatment strategies and the extension of lifespan for patients. Nowadays, an increasing number of studies have implicated that circRNAs have great potential as biomarkers for PC diagnosis and prognosis (Table 2). To be specific, owing to the special splicing mode of circRNAs, most circRNAs are highly conserved in various species 156. Moreover, as a consequence of its unique covalent closed-loop structure, circRNA is resistant to RNA exonuclease or RNase R and has a longer half-life in tissues and plasma. On this basis, it is more stable than linear RNA, which not only is instrumental in the circRNA accumulation, but also is more easily to be detected in cells, tissues or body fluids 157. Apart from that, the expression profiles of circRNAs exhibit cell type specificity, tissue specificity, or developmental stage specificity 158. Furthermore, circRNAs are widely expressed and can be detected in multiple species (such as yeast, plants, fungi, mice, rats, monkeys, fruit flies, humans, and many other organisms). Last but not least, other than solid tissues, circRNAs are also expressed in exosomes, blood, saliva, and urine, which can be employed for non-invasive diagnosis 159.

CircSTX6 has been demonstrated to be frequently upregulated in PDAC. Moreover, the up-regulation of circSTX6 expression in tumor tissues is positively correlated with tumor size and N stage. On this basis, circSTX6 can serve as a potential biomarker for the management of PDAC 79. Zhao *et al.* uncovered that circRNF13 expression was highly abundant in PC tissues and was positively correlated with T stage, N stage, M stage and American Joint Committee on Cancer stage, which might be a potential prognostic indicator 120. Apart from that, circ-MTHFD1L is a GEM resistance-associated circRNA that is conspicuously heightened and stably expressed in PDAC tissues. And the high circ-MTHFD1L expression level is dramatically bound up with chemotherapy resistance and undesirable prognosis of PDAC patients, which may be a new potential molecular marker for GEM resistance 105. Circ_001569 is not only highly expressed in tissues and plasma of PC patients, but also is positively associated with lymphatic metastasis, clinical stage, and venous invasion. Moreover, multivariate Cox regression analyses validated that circ_001569 was an independent prognostic indicator for overall survival rates of PC patients. As evidently demonstrated by operating characteristic (ROC) curve analysis, the area under curve of plasma circ_001569 was 0.716 with a sensitivity and specificity of 62.76% and 74.29%, respectively 70. Likewise, Kaplan-Meier analysis for circNEIL3 revealed that overall survival of PC patients with high expression of circNEIL3 was strikingly shortened. Univariate and multivariate Cox regression analysis collectively revealed that the circNEIL3 expression level was independent prognostic factors for PDAC patients, as were tumor size and TNM stage 81.

On the contrary, some circRNAs are down-regulated in PC, which is associated with a favourable prognosis. For instance, circACTR2 is not only poorly expressed in PC, but also is bound up with overall survival and pathological grade. As further revealed by univariate and multivariate Cox regression analysis, circACTR2 expression levels is independent prognostic factors for PC patients 106. Kong *et al.* held a standpoint that circNFIB1 expression was dramatically decreased in PDAC tissues, and was negatively correlated with lymphatic metastasis and TMN stage 72. Another study exhibited that tumor suppressor circ_0092367 expression was decreased in PC, and attenuated PC progression. Low circ_0092367 expression is tightly connected with shorter survival time 109.

What deserves mentioning is that circRNAs can be detected either in tumor tissues, or in exosomes, blood, saliva, and urine. For instance, circ-PDE8A is upregulated in PDAC tissues and plasma exosomes. Expression level of circ-PDE8A in plasma exosomes is bound up with lymphatic invasion, TNM stage and a disappointing survival rate of PDAC patients. Moreover, exosomal circ-PDE8A may be a useful marker of PDAC diagnosis or progression 74. In comparison, exosomal circ-IARS secreted by PC cells in plasma can be utilized as an indicator for early diagnosis and prognostic prediction in PDAC. For the critical role of circ-IARS in regulating endothelial monolayer permeability, it can be employed as a therapeutic target 73. Wang *et al.* pointed out that exosomal hsa_circ_0012634 was not only isolated from serums and cell culture supernatant, but also was dramatically downregulated in PDAC 112. What's more, ROC curve analysis about hsa_circ_0012634 showed the Area Under the ROC curve value was 0.868, suggesting that hsa_circ_0012634 could be serve as an indicator for distinguishing the population of PDAC patients and healthy controls. Other than exosomal circRNA secreted by tumor cells, exosomal circRNA can also be produced by other cells in the tumor microenvironment. For instance, exosomal circ_0030167 derived from BM-MSCs is decreased in PC serums and cells 119. Furthermore, Lin and colleagues first adopted RNA sequencing to screen for hypoxia-induced exosomal circRNAs in PC, and subsequently noticed that circPDK1 was highly expressed in PC tissues and serum exosomes, which may support a novel diagnostic biomarker for early PC patients 111. These fundamental studies noticeably illustrate that circRNAs have potential as diagnostic and prognostic biomarkers for PC. Nonetheless, it's critical to collect more samples with more typical features to further confirm the reproducibility and specificity of circRNAs detection. Only under such circumstance can they be applied to cancer diagnosis and prognosis evaluation.

### 5.2 Therapeutic targets

For the time being, a multitude of studies have been carried out to explore molecular drugs that regulate gene level based on technologies such as siRNA, antisense oligonucleotides 160 and Clustered Regularly Interspaced Short Palindromic Repeats (CRISPR)/CRISPR-associated protein system 161. Fortunately, numerous drugs originating from these findings have been applied in clinic. Meanwhile, a mushrooming number of domestic and international studies have implicated that circRNAs have been certified to conduct pivotal roles in the occurrence and development of PC. As a consequence, these significant findings provide adequate evidence to support the conjecture that circRNAs may become therapeutic targets for PC. Guo and colleagues analyzed microarray data in PDAC patients and found therapeutic target of PDAC circBFAR notably upregulated in PDAC. CircBFAR knockdown suppresses the proliferation, migration, and invasion of PDAC cells *in vitro* and *in vivo* 98.

Apart from that, the aberrantly expression of circ-0005105 in PDAC can modulate the expression of collagen type XI alpha 1, which is conspicuously correlated with undesirable prognosis of PDAC, thereby lowering the tumorigenicity and metastasis of PDAC cells 96. CircPTPRA, which is positively associated with lymph node invasion and dissatisfactory prognosis, strkingly pushes ahead the migration, invasion, proliferation and EMT of PDAC *in vitro* and* in vivo* 95. Furthermore, circ-ASH2L facilitates PDAC invasion, proliferation and angiogenesis by aberrantly activating Notch signaling pathway, and may be a therapeutic target of PDAC 121. Intriguingly, circEYA3 regulates energy production via ATP synthesis to accelerate PDAC progression, and circEYA3 may be an efficient molecular therapeutic target in PDAC 114. It's critical to note that the hypoxic-induced exosomal circZNF91 can heighten deacetylation-dependent stability of HIF-1α protein. knockdown of circZNF91 retarded glycolysis PC cells 103. Possible therapeutic target circUBAP2 can stabilize the expressions of CXC motif chemokine receptor type 4 and Zinc-finger E-box-binding homeobox 1, hold back antigen presentation, lower the infiltration and function of immune cells, and ultimately facilitate immune escape mechanisms of PC 126. CircACTR2 overexpression retards GEM resistance in PC through activating the phosphatidylinositol-3-kinase/AKT signaling pathway 106.

In summary, the above findings clearly demonstrate that circRNAs play central roles in PC development, tumor microenvironment composition, metabolic changes, immune escape, and chemotherapy resistance. These studies persuasively prove the conjecture that the potential of circRNAs as therapeutic targets or new drugs for PC, but these viewpoints are mostly based on theoretical assumptions and basic experiments. On this basis, it is extremely significant to conduct more basic research and clinical studies to validate these ideas.

## 6. Summary and Prospects

As already confirmed by extensive studies, circRNAs play key regulatory roles in the occurrence and development of various tumors, including PC. As described in this review, circRNAs have been revealed to be involved in dissimilar biological processes of PC and have the potential as biomarkers and therapeutic targets for the diagnosis and prognosis of PC. Nonetheless, it is worthy of mentioning that the current research on circRNAs in PC is still in the early stage, and relevant studies have some limitations. First and foremost, there is no unified standard for the naming of circRNA at present, and unifying the naming rules of circRNA is beneficial for sharing research findings and accelerating the research progress of circRNA. Aside from that, the majority of current studies merely concentrate on the downstream molecular mechanism of circRNAs, while their attention is less fixed on how to explore its upstream molecular mechanism. For instance, the mechanisms of biogenesis and degradation of circRNAs as well as how circRNAs are exported from the nucleus to the cytoplasm. In such case, it's crucial to carry out further elucidation of the upstream molecular mechanism of circRNAs, which is evidently advantageous for us to deepen the understanding of circRNAs. Additionally, numerous studies have implicated that circRNAs regulate various biological processes of PC primarily by acting as miRNA sponges, while there are few studies on their biological functions by binding proteins and regulating gene transcription. Confusingly, there is currently no research on PC-specific circRNAs encoded peptides. Apart from that, the expression abundances of most circRNAs are much lower than that of miRNAs. As a result, it is essential to further elucidate other mechanisms by which circRNAs playing roles. To go it further, the detection of circRNAs at present are predominantly conducted in clinical tissue samples, and their expression profiles in various body fluid samples should also be taken seriously. Simultaneously, circRNAs in various clinical samples should be quantified, and more attention should be immersed in the specific expressions of circRNAs in PC. Finally, the strategy based on circRNAs for the treatment of PC is still in its infancy. It is paramount to figure out how to accurately overexpress or knock down circRNAs, how to accurately transport circRNAs to tumor tissues, and how to avoid possible immune rejection during delivery. To cope well with these problems will not only provide a brand-new viewpoint for clarifying the prominent role of circRNAs in PC biology, but also is advantageous for materializing the transformation of circRNAs from basic research to clinical application.

## Supplementary Material

Supplementary table.

## Figures and Tables

**Table 1:** Databases regarding circRNA

Database	Description
circBank	Search the genomic linear structure, mature sequences, expression patterns across different tissues or developmental stages, miRNA regulatory sites, N6-methyladenosine (m6A) sites, internal ribosome entry site (IRES), coding potential and open reading frames (ORFs).
circBase	Search the genomic position, spliced length, gene symbol, best-matching transcript and circRNA study.
TransCirc	Search the genomic position, gene symbol, m6A sites, IRES, ORFs, ribosome profiling and mass spectrometry data.
circRNADb	Search the genomic position, spliced length, gene symbol, best-matching transcript, sample, IRES and ORFs.
Circular RNA Interactome	Search the genomic position, best-matching transcript, mature sequences, RBPs and miRNA regulatory sites, divergent primers and siRNAs.
CIRCpedia	Search the genomic position, gene symbol, sample and conservation analysis
CircNet	Search the genomic position, gene symbol, RBPs and miRNA regulatory sites, ORFs
circAtlas	Search the genomic position, gene symbol, the second RNA structure, sample, conservation analysis, RBPs and miRNA regulatory sites, IRES and ORFs.

**Table 2:** CircRNAs act as potential diagnostic and prognostic biomarkers in pancreatic cancer

circRNA	Expression	Diagnostic	Prognostic	Clinicopathological parameters						Ref.
				Pathological stage	Tumor stage	Lymph node status	Distant metastasis	TNM stage	Other	
circ-LDLRAD3	Up	Yes	Yes	/	/	Yes	Yes	/	Vascular invasion	87
hsa_circ_0007367	Up	/	Yes	Yes	/	Yes	/	/	/	90
circSTX6	Up	/	Yes	Yes	/	Yes	/	/	/	79
circNEIL3	Up	/	Yes	/	/	/	/	/	Vascular invasion, Nerve invasion	81
circ_0087502	Up	/	Yes	Yes	Yes	Yes	/	/	/	107
hsa_circ_0046523	Up	/	Yes	Yes	Yes	Yes	/	Yes	/	129
circMYO1C	Up	/	/	/	/	Yes	/	Yes	/	130
circCUL2	Up	Yes	Yes	/	/	Yes	/	Yes	/	100
circRNF13	Up	/	Yes	/	Yes	Yes	Yes	/	/	120
circ-ASH2L	Up	/	Yes	/	/	Yes	/	Yes	/	121
circRNA_000684	Up	/	Yes	Yes	/	Yes	Yes	Yes	/	123
circSLIT2	Up	/	Yes	/	/	Yes	/	Yes	/	110
circ_001569	Up	Yes	Yes	/	/	Yes	/	Yes	Vascular invasion	70
circBFAR	Up	/	Yes	/	/	/	/	Yes	/	98
circ-0005105	Up	/	Yes	/	Yes	Yes	/	Yes	Vascular invasion	96
circPTPRA	Up	/	Yes	/	/	Yes	/	/	/	95
circ_0030235	Up	/	Yes	/	Yes	Yes	/	/	/	65
circ_0005273	Up	Yes	Yes	/	/	Yes	Yes	/	/	77
Exosomal hsa_circ_0012634	Down	/	/	/	Yes	/	/	/	/	112
hsa_circRNA_001587	Down	/	Yes	Yes	/	Yes	/	/	/	124
circ_0092367	Down	/	Yes	Yes	/	Yes	/	/	/	109
circ_0013587	Down	/	Yes	Yes	Yes	Yes	/	/	/	108
circNFIB1	Down	/	/	/	/	Yes	/	Yes	/	72
circACTR2	Down	/	Yes	Yes	/	/	/	/	/	106
hsa_circ_0001649	Down	/	Yes	Yes	Yes	/	/	/	/	50
Exosomal circ-PDE8A	Up	/	Yes	/	Yes	Yes	/	Yes	/	74
Exosomal circPDK1	Up	/	Yes	Yes	Yes	Yes	Yes	/	/	111
Exosomal circ-IARS	Up	/	Yes	/	/	/	Yes	/	/	73
hsa_circ_0071036	Up	Yes	Yes	/	/	Yes	/	/	PET-CT SUV max value	17
circATG7	Up	Yes	Yes	/	Yes	Yes	/	/	/	19
circEIF3I	Up	/	Yes	/	Yes	Yes	/	/	/	20
circRHOBTB3	Up	/	Yes	/	Yes	/	/	/	Vascular invasion	32
circRHOT1	Up	/	/	/	/	Yes	/	/	/	57
circ_0007534	Up	/	Yes	/	Yes	Yes	/	/	/	63
circ_0092314	Up	/	Yes	/	/	Yes	/	Yes	/	84
circ-MTHFD1L	Up	/	Yes	Yes	/	/	/	/	CA19-9	105
hsa_circ_0000069	Up	Yes	Yes	/	Yes	/	Yes	/	/	59
circ-ADAM9	Up	/	Yes	/	/	Yes	Yes	Yes	/	99
circEYA3	Up	/	Yes	/	/	/	/	Yes	CA19-9	114
circPCDH10	Up	/	Yes	Yes	Yes	/	Yes	Yes	/	49
circEIF6	Up	Yes	Yes	/	/	/	/	Yes	/	61
circ_0128846	Up	/	/	/	/	/	/	Yes	/	62
circNEK6	Up	Yes	Yes	/	/	Yes	/	/	/	69
circCCT3	Up	/	Yes	/	/	Yes	/	Yes	Peritoneal metastasis, Vascular invasion	66
hsa_circ_0074298	Up	Yes	/	Yes	Yes	Yes	/	/	/	56
ciRS-7	Up	/	/	/	/	Yes	/	/	Vascular invasion	88
circ_0075829	Up	/	/	/	Yes	Yes	/	/	/	92
circ_0013912	Up	/	/	/	/	Yes	/	Yes	/	64
circARFGEF2	Up	/	Yes	/	/	Yes	/	/	/	71
