# Supplementary material for: Circular RNA in Pancreatic Cancer: Biogenesis, Mechanism, Function and Clinical Application
Source: Int J Med Sci. 2025 Feb 28;22(7):1612–29. doi: 10.7150/ijms.107773 (PMC11905278; doi:10.7150/ijms.107773)
Supplement: Supplementary file 1 — Supplementary table. [file ijmsv22p1612s1.pdf]

Supplementary table 1 Mechanisms and Functions of circRNAs in PC.

circRNA	Mechanism	Biological function	Ref.
circ_100782	MiRNA sponge	Proliferation	[48]
circPCDH10	MiRNA sponge	Proliferation	[49]
circANAPC7	MiRNA sponge	Proliferation	[15]
circRHOBTB3	MiRNA sponge	Proliferation and autophagy	[30]
circATG7	MiRNA sponge and protein scaffold	Proliferation, autophagy and metastasis	[18]
hsa_circ_0001649	/	Proliferation and apoptosis	[50]
circDUSP22	MiRNA sponge	Proliferation and apoptosis	[51]
circFOXK2	MiRNA sponge and protein scaffold	Proliferation, apoptosis and metastasis	[52]
circFARSA	/	Proliferation, apoptosis and metastasis	[53]
circSFMBT1	MiRNA sponge	Proliferation, apoptosis and metastasis	[54]
circRNA_000864	MiRNA sponge	Proliferation, apoptosis and metastasis	[55]
hsa_circ_0074298	MiRNA sponge	Proliferation, apoptosis and metastasis	[56]
circRHOT1	MiRNA sponge	Proliferation, apoptosis and metastasis	[57]
circ-0050102	MiRNA sponge	Proliferation, apoptosis and metastasis	[58]
hsa_circ_0000069	MiRNA sponge	Proliferation, apoptosis and metastasis	[59]
hsa_circ_0006215	MiRNA sponge	Proliferation, apoptosis and metastasis	[60]
hsa_circ_0071036	MiRNA sponge	Proliferation, apoptosis and metastasis	[16]
circEIF6	MiRNA sponge	Proliferation, apoptosis and metastasis	[61]
circ_0128846	MiRNA sponge	Proliferation, apoptosis and metastasis	[62]
circ_0007534	MiRNA sponge	Proliferation, apoptosis and metastasis	[63]
circ_0013912	MiRNA sponge	Proliferation, apoptosis and metastasis	[64]
circ_0030235	MiRNA sponge	Proliferation, apoptosis and metastasis	[65]
circCCT3	MiRNA sponge	Proliferation, apoptosis and metastasis	[66]
circFGFR1	MiRNA sponge	Proliferation, apoptosis and metastasis	[67]
circUHRF1	MiRNA sponge	Proliferation, apoptosis and metastasis	[68]
circNEK6	MiRNA sponge	Proliferation, apoptosis and metastasis	[69]
circ_001569	/	Proliferation, apoptosis and	[70]

		metastasis	
circARFGEF2	MiRNA sponge	Metastasis	[71]
circNFIB1	MiRNA sponge	Metastasis	[72]
circ-IRAS	MiRNA sponge	Metastasis	[73]
circ-PDE8A	MiRNA sponge	Metastasis	[74]
circRNA_0047744	MiRNA sponge	Metastasis	[75]
circ_0001666	MiRNA sponge	Metastasis	[76]
circ_0005273	Regulators of gene splicing and transcription	Proliferation and metastasis	[77]
circRTN4	MiRNA sponge and binding RBP	Proliferation and metastasis	[78]
circSTX6	MiRNA sponge and binding RBP	Proliferation and metastasis	[79]
circEIF3I	MiRNA sponge and protein scaffold	Proliferation and metastasis	[20]
circ_001859	MiRNA sponge	Proliferation and metastasis	[80]
circNEIL3	MiRNA sponge	Proliferation and metastasis	[81]
hsa_circ_0000994	MiRNA sponge	Proliferation and metastasis	[18]
circZFR	MiRNA sponge	Proliferation and metastasis	[82]
circSEC24A	MiRNA sponge	Proliferation and metastasis	[83]
circ_0092314	MiRNA sponge	Proliferation and metastasis	[84]
circ-STK39	MiRNA sponge	Proliferation and metastasis	[85]
circ_0008768	MiRNA sponge	Proliferation and metastasis	[86]
circ-LDLRAD3	MiRNA sponge	Proliferation and metastasis	[87]
ciRS-7	MiRNA sponge	Proliferation and metastasis	[88]
circ_0000106	MiRNA sponge	Proliferation and metastasis	[47]
circ_103809	MiRNA sponge	Proliferation and metastasis	[89]
hsa_circ_0007367	MiRNA sponge	Proliferation and metastasis	[90]
circCDR1	MiRNA sponge	Proliferation and metastasis	[91]
circ_0075829	MiRNA sponge	Proliferation and metastasis	[92]
hsa_circ_0006117	MiRNA sponge	Proliferation and metastasis	[93]
circPVRL3	MiRNA sponge	Proliferation and metastasis	[94]
circPTPRA	MiRNA sponge	Proliferation and metastasis	[95]
circ-0005105	MiRNA sponge	Proliferation and metastasis	[96]
circ-MYBL2	MiRNA sponge	Proliferation and metastasis	[97]
circBFAR	MiRNA sponge	Proliferation and metastasis	[98]
circ-ADAM9	MiRNA sponge	Proliferation and metastasis	[99]
circCUL2	MiRNA sponge	Proliferation and metastasis	[100]
hsa_circ_0007401	MiRNA sponge	Chemotherapy resistance	[101]
hsa_circ_0007919	Regulators of gene splicing and transcription	Chemotherapy resistance	[102]
circZNF91	MiRNA sponge	Glycolysis and chemotherapy resistance	[103]
circFARP1	MiRNA sponge and binding RBP	Proliferation and chemotherapy resistance	[104]
circ-MTHFD1L	MiRNA sponge	Proliferation and chemotherapy resistance	[105]
circACTR2	MiRNA sponge	Proliferation, apoptosis and	[106]

		chemotherapy resistance	
circ_0087502	MiRNA sponge	Proliferation, metastasis and chemotherapy resistance	[107]
circ_0013587	MiRNA sponge	Proliferation, metastasis and chemotherapy resistance	[108]
circ_0092367	MiRNA sponge	Proliferation, metastasis and chemotherapy resistance	[109]
circHIPK3	MiRNA sponge	Proliferation, apoptosis, metastasis and chemotherapy resistance	[30]
circSLIT2	MiRNA sponge	Proliferation and glycolysis	[110]
circPDK1	MiRNA sponge and protein scaffold	Proliferation, metastasis and glycolysis	[111]
hsa_circ_0012634	MiRNA sponge	Proliferation, apoptosis and glycolysis	[112]
circ_03955	MiRNA sponge	Proliferation, apoptosis and glycolysis	[113]
circEYA3	MiRNA sponge	Proliferation, metastasis and glycolysis	[114]
circ_0072088	MiRNA sponge	Proliferation, apoptosis, metastasis and glycolysis	[115]
circ_0099999	MiRNA sponge	Proliferation, apoptosis, metastasis and glycolysis	[116]
circ-MBOAT2	MiRNA sponge	Proliferation, apoptosis, metastasis and glutamine catabolism	[117]
circRREB1	Binding RBP	Glycolysis and stemness	[118]
circ_0030167	MiRNA sponge	Proliferation, metastasis and stemness	[119]
circRNF13	MiRNA sponge	Proliferation, metastasis, glycolysis and angiogenesis	[120]
circ-ASH2L	MiRNA sponge	Proliferation, metastasis and angiogenesis	[121]
hsa_circ_0014784	MiRNA sponge	Proliferation, metastasis and angiogenesis	[122]
circRNA_000684	MiRNA sponge	Proliferation, metastasis and angiogenesis	[123]
hsa_circRNA_001587	MiRNA sponge	Proliferation, metastasis and angiogenesis	[124]
circ_0000284	MiRNA sponge	Proliferation, apoptosis, metastasis and angiogenesis	[125]
hsa_circ_0050102	MiRNA sponge	Proliferation, apoptosis, metastasis and angiogenesis	[58]
circ-UBAP2	MiRNA sponge	Immune escape	[126]
circ_0000977	MiRNA sponge	Immune escape	[127]
circPTPN22	MiRNA sponge and protein scaffold	Proliferation and immune escape	[128]
hsa_circ_0046523	MiRNA sponge	Proliferation, metastasis and immune escape	[129]

circMYO1C	Binding RBP	Proliferation, metastasis and immune escape	[130]
circ_0018909	MiRNA sponge	Proliferation, apoptosis, metastasis and immune escape	[131]
hsa_circ_0006790	Binding RBP	Proliferation, apoptosis, metastasis and immune escape	[132]
circ_0058058	MiRNA sponge	Proliferation, metastasis, angiogenesis and immune escape	[133]
circRNA_102049	MiRNA sponge	Proliferation, apoptosis, metastasis and inflammatory response	[134]
